# Minimal Access (Endoscopic and Robotic) Breast Surgery in the Surgical Treatment of Early Breast Cancer—Trend and Clinical Outcome From a Single-Surgeon Experience Over 10 Years

**DOI:** 10.3389/fonc.2021.739144

**Published:** 2021-11-19

**Authors:** Hung-Wen Lai, Shou-Tung Chen, Ying-Jen Lin, Shih-Lung Lin, Ching-Min Lin, Dar-Ren Chen, Shou-Jen Kuo

**Affiliations:** ^1^ Endoscopic and Oncoplastic Breast Surgery Center, Department of Surgery, Changhua Christian Hospital, Changhua, Taiwan; ^2^ Division of General Surgery, Department of Surgery, Changhua Christian Hospital, Changhua, Taiwan; ^3^ Comprehensive Breast Cancer Center, Department of Surgery, Changhua Christian Hospital, Changhua, Taiwan; ^4^ Minimal Invasive Surgery Research Center, Department of Surgery, Changhua Christian Hospital, Changhua, Taiwan; ^5^ Department of Surgery, Kaohsiung Medical University, Kaohsiung, Taiwan; ^6^ Division of Breast Surgery, Department of Surgery, Yuanlin Christian Hospital, Yuanlin, Taiwan; ^7^ Department of Surgery, School of Medicine, National Yang Ming University, Taipei, Taiwan; ^8^ Department of Surgery, School of Medicine, Chung Shan Medical University, Taichung, Taiwan; ^9^ Department of Surgery, Chang Gung University College of Medicine, Taoyuan City, Taiwan; ^10^ Division of General Surgery, Department of Surgery, Kaohsiung Chang Gung Memorial Hospital, Kaohsiung, Taiwan; ^11^ Tumor Center, Changhua Christian Hospital, Changhua, Taiwan; ^12^ Division of Plastic and Reconstructive Surgery, Department of Surgery, Changhua Christian Hospital, Changhua, Taiwan

**Keywords:** endoscopy-assisted breast surgery (EABS), conventional breast surgery (CBS), endoscopy-assisted breast conserving surgery (E-BCS), endoscopic assisted nipple sparing mastectomy (E-NSM), robotic assisted nipple sparing mastectomy (R-NSM), single-port three-dimensional videoscope-assisted endoscopic nipple sparing mastectomy (3D E-NSM)

## Abstract

**Objective:**

Endoscopic assisted breast surgery (EABS) or robotic assisted breast surgery (RABS) performed through minimal axillary and/or peri-areolar incisions has become the representative of minimal access breast surgery (MABS). We report the trend and clinical outcome of MABS for treatment of breast cancer.

**Methods:**

Information on patients who underwent breast cancer operation by the principal investigator during the period of 2011 to 2020 was collected from a single institute for analysis. The clinical outcome, trend, and cost of MABS were analyzed and compared with conventional breast surgery (CBS).

**Results:**

A total of 824 breast cancer patients operated by a single surgeon were enrolled in this study: 254 received CBS and 570 received MABS, namely, 476 EABS and 94 RABS. From 2011 to 2020, the number of MABS performed annually has shown an increasing trend. Compared with CBS, MABS such as breast conserving surgery and nipple sparing mastectomy (NSM) have effectively reduced wound scar length. Since the sequential uprise from conventional NSM (C-NSM), dual-axillary-areolar-incision two dimensional (2D) endoscopic assisted NSM (E-NSM), single-axillary-incision E-NSM, robotic assisted NSM (R-NSM), and single-port 3D E-NSM, the development of minimal access mastectomies increasingly paralleled with NSM. The operation time of various MABS decreased significantly and showed no statistical difference compared with CBS. R-NSM was associated with highest cost, followed by 3D E-NSM, E-NSM, and C-NSM. The positive surgical margin rate and local recurrence rate of MABS and CBS were not statistically different.

**Conclusion:**

MABS showed comparable clinical outcome and preliminary oncologic safety as CBS and has been increasingly performed as the surgical treatment of breast cancer, especially minimal access NSM.

## Introduction

Minimal invasive/access surgery has become the mainstream of surgical practice in recent decades (1–3). Endoscopic assisted breast surgery (EABS) (4–6) or robotic assisted breast surgery (RABS) (7–9) performed through minimal axillary and/or peri-areolar incisions has become the representative of minimal access breast surgery (MABS) (10). MABS, either EABS or RABS, has been performed in breast conserving surgery (BCS) (4, 11, 12), mastectomy [mainly nipple sparing mastectomy (NSM) or skin sparing mastectomy (SSM) in some conditions] (7, 8, 13–17), and harvest of autologous flaps [latissimus dorsi flap (LDF) (18–20), omentum flap (21–23), or abdominal flap (14, 24, 25)]. Due to the wide spread of the breast screening program, the number of early breast cancers diagnosed increased dramatically and so is the reported use of MABS in literature around the world (15, 26, 27).

The advantages of MABS (EABS or RABS) include shortening of operation scar while hiding it in inconspicuous locations, which optimize aesthetic outcome and patients’ satisfaction (9, 11, 15, 17). However, the widespread use of MABS in the management of breast cancer is yet to be fully accepted; objections include limited working space, superficial nature of breast lesion, and relative low morbidity of breast surgery (28). Drawbacks include longer operation time, more instruments needed, and higher medical cost (9, 17, 29) Of utter importance, the long-term oncologic outcome is rarely reported (5, 26) and not yet confirmed by large randomized trials.

At our institution, we started performing EABS (4, 13–15, 26, 30, 31) for breast cancer treatment since 2010 and developed RABS (9, 17–19, 32–34) in 2017. After 10 years of work in the field of MABS, much experience and accumulated results of its applications in breast cancer were obtained. The development and trend of MABS (EABS or RABS) versus conventional breast surgery (CBS) in the past decade were evaluated from the principal investigator’s personal perspective. The clinical outcome, complications, and cost of MABS versus CBS were also analyzed and reported.

## Materials and Methods

### Patients

**Figure 1:**
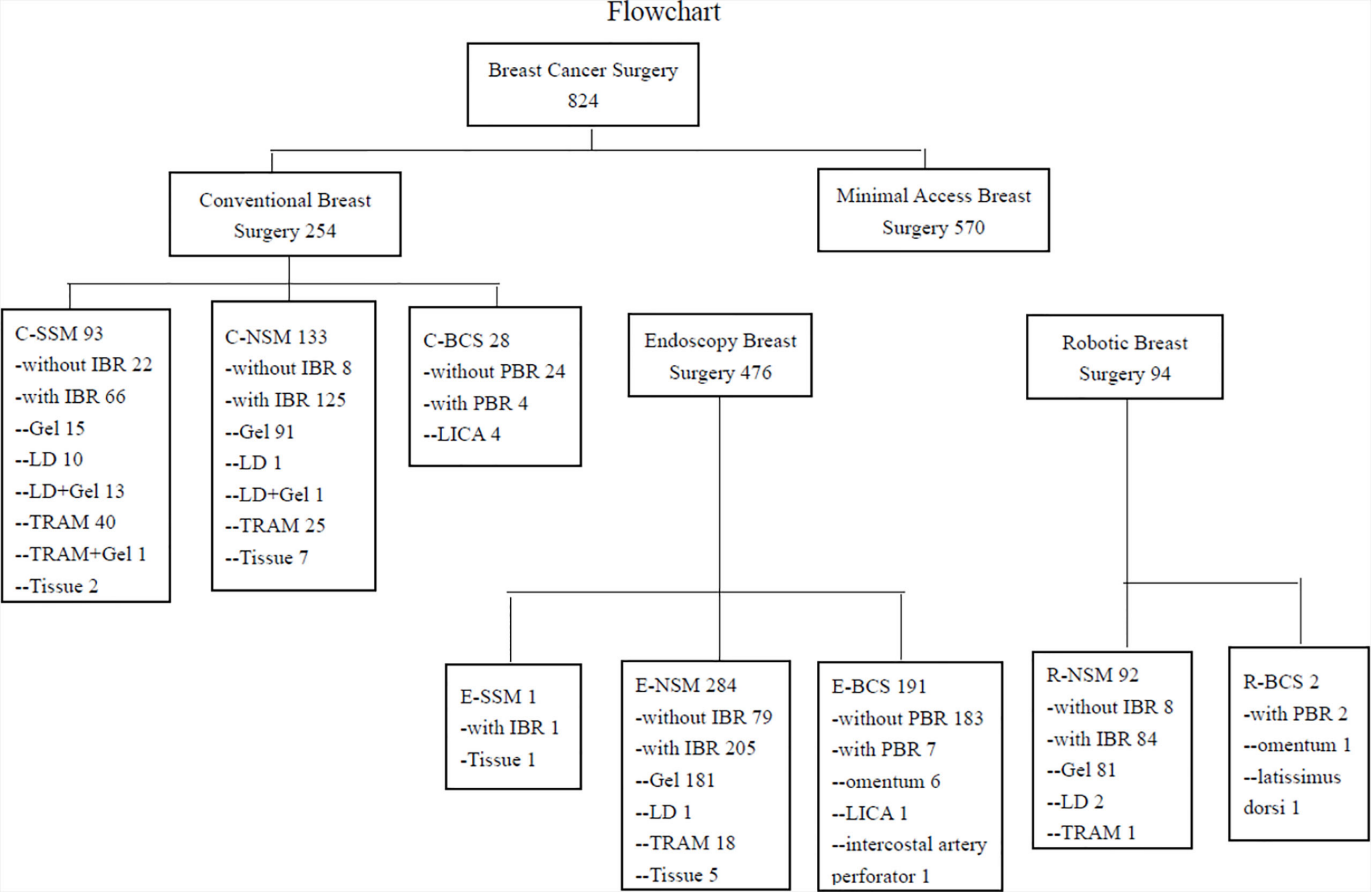
Flow chart of patients’ management in the current study. C-SSM, conventional skin sparing mastectomy; C-NSM, conventional nipple sparing mastectomy; C-BCS, conventional breast conserving surgery; PBR, partial breast reconstruction; LICA, lateral intercostal artery perforator flap; IBR, immediate breast reconstruction; LD, latissimus dorsi flap; TRAM, transverse abdominal rectus muscular flap; Tissue, tissue expander; E-SSM, endoscopic skin sparing mastectomy; E-NSM, endoscopic nipple sparing mastectomy; E-BCS, endoscopic breast conserving surgery; R-NSM, robotic nipple sparing mastectomy; R-BCS, robotic breast conserving surgery.

Patients who received CBS or MABS (EABS or RABS) for breast cancer by the principal investigator (H-WL) from January 2011 to December 2020 were retrieved from a prospectively collected endoscopic and robotic assisted breast surgery database at Changhua Christian Hospital (CCH) in Taiwan. Patient selection and enrollment process are shown in **Figure 1**.

The data collection included clinicopathologic characteristics of patients, type of surgery, operative time, blood loss, length of hospital stays, recurrence, and survival status at last follow-up. All data were collected from chart review by specially trained nurses and subsequently confirmed by the principal investigator (H-WL). The study was approved by the Institutional Review Board of the CCH (CCH IRB No. 141224, 170806, and 190414). Written informed consent pertaining to the use of clinical records was obtained from each participant. This current report included photos of several patients who had agreed and signed the consent for publication of their pictures.

### Indication for MABS (EABS or RABS)

Pre-operative sonography, mammography, and/or magnetic resonance imaging (MRI) were used to determine the eligibility of patients for MABS (EABS or RABS). Liver sonography, chest x-ray, and whole-body bone scan were used to exclude the possibility of distant metastasis. Indications for EABS or RABS include early-stage breast cancer (ductal carcinoma *in situ*, stage I, II or IIIA), a tumor size less than 3 cm (for BCS) or no larger than 5 cm (for mastectomy), absence of apparent multiple lymph nodes metastases, and absence of skin or chest wall invasion.

Patients for whom EABS or RABS was contraindicated include those with inflammatory breast cancer, breast cancer with chest wall or skin invasion, locally advanced breast cancer, breast cancer with extensive axillary lymph node metastasis (stage IIIB or later), and severe comorbidities, such as heart disease, renal failure, liver dysfunction, and poor performance status as assessed by their primary physicians. The inclusion and exclusion criteria were based on previous studies (11, 13–15, 27, 34) as well as current breast cancer treatment guidelines.

### Minimal Access Endoscopic or Robotic Breast Surgery Technique

**Figure 2:**
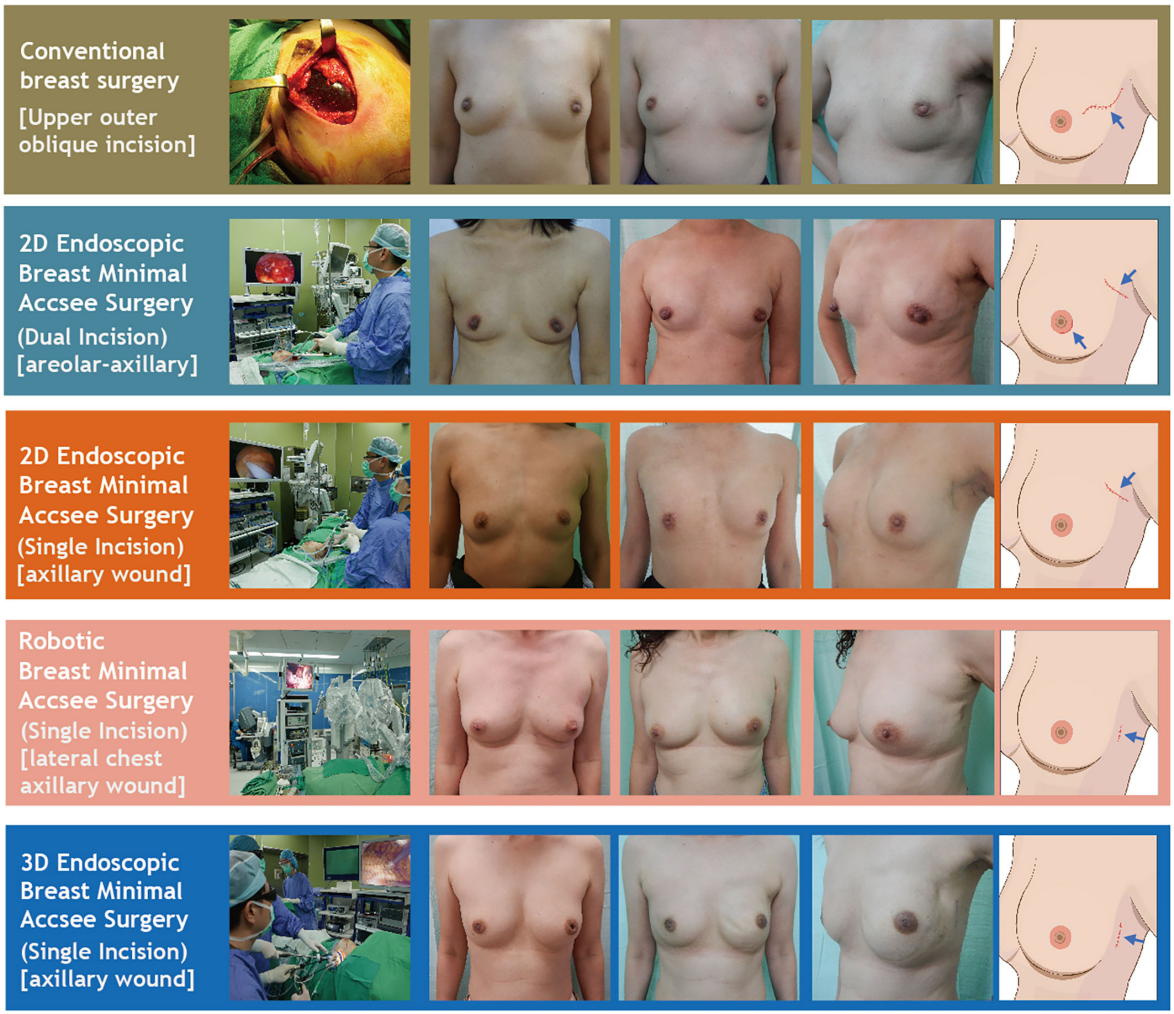
Various surgical technique of nipple sparing mastectomy (NSM). This figure showed various NSM techniques used in the current study, including conventional breast surgery, 2D endoscopic breast surgery (dual-areolar-axillary-incision), 2D endoscopic single-axillary-incision breast surgery, robotic assisted breast surgery, and single-port 3D assisted breast surgery. Photos are shown in each category including pre-operative frown view, post-operative front view, post-operative lateral view, and cartoon illustration of scar location.

Details of the surgical technique used for EABS (11, 13–15, 31) or RABS (18, 19, 27, 32, 34) in the current study have been described previously. Endoscopic assisted or robotic assisted BCS and mastectomy (with or without breast reconstructions) were common MABS or aesthetic scar-less mastectomy (35) procedures for surgical treatments of breast cancers. Common incisions for MABS include axillary and/or areolar incisions *via* either dual incisions or single areolar, axillary, or lateral chest incision as per case indicated (**Figure 2** and **Supplementary Figure 1**). Whether breast reconstructions were performed immediately or at a later time is decided with patients and physicians’ shared decision making. Breast reconstructions after minimal access mastectomy (or aesthetic scar-less mastectomy^35^) can be performed by using an implant (cohesive gel implants or tissue expander) (13, 15, 34) or autologous tissue with latissimus dorsi (LD) flap (18, 19) or abdominal flap (14, 24, 25) (**Figure 2** and **Supplementary Figure 1**).

### Clinical Outcome of Minimal Access Breast Surgery

Peri-operative parameters, such as operation time, blood loss, complications, and hospital stay, of CBS, EABS, and RABS were analyzed and compared. Complications were also graded with the Clavien-Dindo classification (36) for severity evaluation. For oncological safety evaluation, we analyzed the rate of positive surgical margin involvement, local regional recurrence, distant metastasis, disease-free survival, and overall survival. Surgical margin involvement was defined as tumor on ink (37). Adjuvant chemotherapy and radiotherapy were given to patients based on recommendations from current breast cancer guidelines. Incidence of recurrence or death due to breast cancer was ascertained at the most recent follow-up, with a cutoff date in April 2021.

### Statistical Analyses

Differences in continuous variables were tested by non-parametric Mann–Whitney *U* test and Kruskal–Wallis test, and are reported as means ± standard deviation (SD). The chi-square test was used for categorical comparisons of data when appropriate. A *p-*value of less than 0.05 was considered of statistical significance; all tests were two-tailed. Differences in cumulative survival were assessed using the log-rank test. All statistical analyses were performed with statistical package SPSS (Version 19.0, SPSS, Chicago) by a statistics personnel (Y-JL).

## Results

During 2011 to 2020, a total of 824 breast cancer patients operated by a single surgeon (H-WL) were enrolled in the current study. Among them, 254 received conventional breast surgeries (CBS) and 570 received MABS. These included 476 EABS and 94 RABS. The characteristics and clinicopathologic parameters of enrolled patients are summarized in **Table 1**, and types of surgeries performed are shown in flow chart and photos (**Figures 1**, **2**). The RABS mainly focusing on R-NSM (92/94) related, and most of the patients received immediate gel implant breast reconstruction (IGBR). The only two robotic assisted BCSs were related to partial breast reconstructions (robotic LD flap harvest or robotic omentum flap harvest). In our current MABS cohort, no case (either EABS nor RABS) required conversion to conventional operation method.

**Table 1:** Clinical presentations of patients enrolled in current study.

	Total (*N* = 824)	Conventional Breast Surgery (*N* = 254)	Minimal Access Breast Surgery (*N* = 570)	*p*-value
Age, years	50.9 ± 10.6	50.9 ± 11.5	51 ± 10.2	0.62
Location				0.81
Right	415 (50.4)	130 (51.2)	285 (50)	
Left	409 (49.6)	124 (48.8)	285 (50)	
Sonogram tumor size, (cm)	2.48 ± 1.7	3.2 ± 2.6	2.2 ± 1	<0.01
Pathology tumor size, (cm)	2.5 ± 2.3	3.4 ± 3.2	2.2 ± 1.8	<0.01
Lymph node metastasis				<0.01
Yes	219 (32.8)	92 (40.9)	127 (24.2)	
No	530 (67.2)	133 (59.1)	397 (75.8)	
Lymph node stage				<0.01
N0	538 (59.9)	137 (60.08)	401 (76.09)	
N1	161 (26.4)	60 (26.32)	101 (19.17)	
N2	45 (9.7)	22 (9.65)	22 (4.36)	
N3	11 (4.0)	9 (3.95)	1 (3.80)	
Stage				<0.01
0	139 (19)	36 (16.3)	103 (20.2)	
I	227 (31.1)	40 (18.1)	187 (36.7)	
II	285 (39)	96 (43.4)	189 (37.1)	
III	71 (9.7)	41 (18.6)	30 (5.9)	
IV	8 (1.1)	8 (3.6)	0 (0)	
Breast operation				<0.01
Total mastectomy	602 (73.18)	226 (88.98)	376 (65.96)	
Partial mastectomy (BCS)	222 (26.82)	28 (11.02)	194 (34.04)	
Lymph node surgery				<0.01
SLNB	550 (75.14)	125 (59.52)	425 (81.42)	
SLNB then ALND	120 (16.39)	39 (18.57)	81 (15.52)	
ALND	62 (8.47)	46 (21.90)	16 (3.07)	
Post-operation Histology				0.80
DCIS	143 (17.6)	38 (15.4)	105 (18.6)	
IDC	137 (16.9)	46 (18.6)	91 (16.1)	
IDC+DCIS	366 (45.1)	110 (44.5)	256 (45.3)	
ILC	11 (1.4)	5 (2)	6 (1.6)	
ILC+LCIS	14 (1.7)	4 (1.6)	10 (1.8)	
LCIS	8 (1)	2 (0.8)	6 (1.1)	
Other	133 (16.4)	42 (17)	91 (16.1)	
Grade				<0.01
I	121 (17.6)	26 (12.5)	95 (19.8)	
II	403 (58.6)	112 (53.9)	291 (60.6)	
III	164 (23.8)	70 (33.7)	94 (19.6)	
ER				0.16
Positive	575 (80.4)	164 (77)	411 (81.9)	
Negative	140 (19.6)	49 (23)	91 (18.1)	
PR				1
Positive	502 (70.6)	150 (70.8)	352 (70.5)	
Negative	209 (29.4)	62 (29.3)	147 (29.5)	
HER-2				<0.01
Positive	168 (26.9)	67 (34.7)	101 (23.4)	
Negative	457 (73.1)	126 (65.3)	331 (76.6)	
Subtype				<0.01
Luminal A	301 (45.7)	65 (32.3)	236 (51.5)	
Luminal B1	155 (23.5)	56 (27.9)	99 (21.6)	
Luminal B2	76 (11.5)	38 (18.9)	38 (8.3)	
HER-2(+)	59 (9)	19 (9.5)	40 (8.7)	
TNBC	68 (10.3)	23 (11.4)	45 (9.8)	
Ki 67				0.03
≦20%	351 (63.8)	100 (56.8)	251 (67.1)	
>20%	199 (36.2)	76 (43.2)	123 (32.9)	
Margin involved				0.39
Yes	22 (3)	9 (4)	13 (2.5)	
No	723 (97)	217 (96)	506 (97.5)	
Follow-up (month)	45.6 ± 32.1, median = 40.7	51.1± 34.1, median = 53.9	43.1 ± 30.9, median = 35.7	<0.01
Recurrence				0.051
Yes	47 (5.7)	21 (8.3)	26 (4.6)	
No	777 (94.3)	233 (91.7)	544 (95.4)	
Metastasis				<0.01
Yes	67 (8.1)	44 (17.3)	23 (4)	
N	757 (91.9)	210 (82.7)	547 (96)	

BCS, breast conserving surgery; DCIS, ductal carcinoma in situ; IDC, infiltrating ductal carcinoma; ILC, infiltrating lobular carcinoma.

### Trend of Minimal Access Breast Surgery

**Figure 3:**
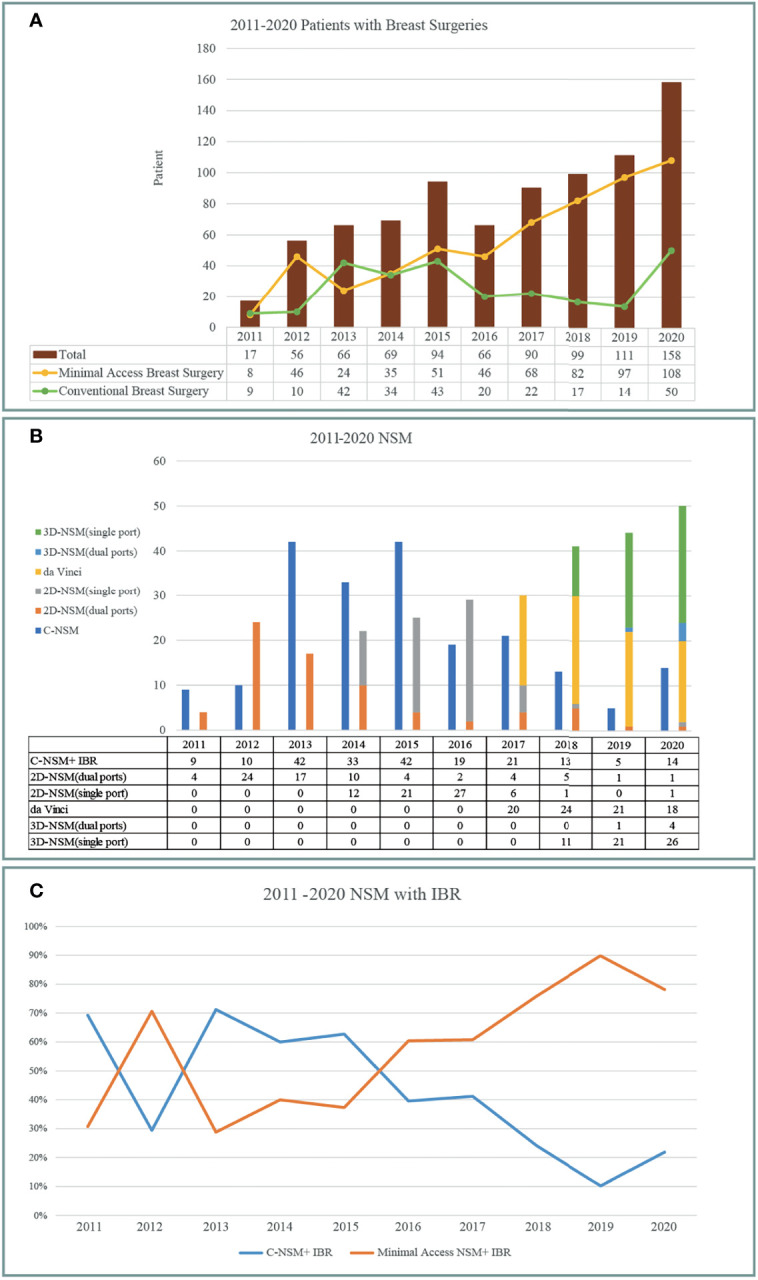
Development and trend of minimal access breast surgery over 2011–2020 from the principal investigator’s perspective. **(A)** Breast cancer operations performed per year according to conventional breast surgery (CBS) and minimal access (including endoscopic or robotic assisted) breast surgery (MABS). MABS increased gradually and became the mainstream of breast cancer operation. **(B)** Various nipple sparing mastectomy (NSM) operations performed per year. Following the sequences of conventional NSM, dual-areolar-axillary-incision 2D endoscopic assisted NSM (E-NSM), single-axillary-incision E-NSM, robotic assisted NSM (R-NSM), and single-port 3D videoscope-assisted E-NSM (3D E-NSM). **(C)** Trend of NSM with immediate breast reconstruction (IBR) during 2011–2020. Minimal access operation gradually became the dominant choices between patients receiving NSM+ IBR.

Breast cancer cases that received CBS or MABS were recorded and plotted from 2011 to 2020 (**Figure 3A**). Number of MABS performed increased annually and became the mainstream choice. Among this cohort of patients, 221 cases received BCS. Twenty-eight received conventional BCS (C-BCS), 191 received endoscopic assisted BCS (E-BCS), and 2 received robotic assisted BCS (R-BCS, **Table 2**). The mean incisional wound length of C-BCS was 8.3 ± 3.1 cm (median 6.5 cm) versus 5.2 ± 0.9 cm in E-BCS (median 5 cm, *p* < 0.01). The operation time of C-BCS versus E-BCS was 101 ± 42 versus 114 ± 43 min (*p* = 0.24), respectively. The median follow-up time of BCS patients was 24.8 months. Comparison between E-BCS and C-BCS showed no statistical difference between mean blood loss, resected specimen weight, hospital stay, positive margin rate, complication, and local recurrence (**Table 2**).

**Table 2:** Comparison of clinical outcome between different breast cancer operations.

	BCS*				NSM with IGBR				
	Total (*N* = 202)	C-BCS (*N* = 24)	E-BCS (*N* = 178)	*p*-value	Total (*N* = 272)	C-NSM (*N* = 84)	E-NSM (*N* = 133)	R-NSM (*N* = 55)	*p*-value
Wound length (cm)	8.1 ± 6.5	8.3 ± 3.1	5.2 ± 0.9	<0.01	5.4 ± 2.5	9.4 ± 4.6	5 ± 0.8	4.1 ± 0.8	*p* < 0.1
	(median 5.5)	(median 6.5)	(median 5)		(median 5)	(median 8)	(median 5)	(median 4)	
Tumor size	Wound length				Wound length				
≤2 cm	4.9 ± 1.6	5.8 ± 2.1	4.4 ± 0.9		5.5 ± 3.0	9.4 ± 3.7	5.0 ± 0.9	4.0 ± 0.8	
	(median 4.75)	(median 5.8)	(median 4.5)		(median 5)	(median 9)	(median 5)	(median 4)	
2.1–5 cm	5.9 ± 2.8	8.5 ± 5.6	5.1 ± 1.3		5.8 ± 3.3	9.5 ± 4.4	5.2 ± 2.6	4.3 ± 0.9	
	(median 5.25)	(median 7.5)	(median 5)		(median 5)	(median 8)	(median 5)	(median 4)	
>5 cm					5.7 ± 2.2	10.9 ± 5.9	5.2 ± 0.4	4.6 ± 0.7	
					(median 5)	(median 10.8)	(median 5)	(median 4.5)	
Surgery time (min)	113 ± 43	101 ± 42	114 ± 43	0.24	199 ± 97	183 ± 101	209 ± 94	192 ± 64	0.12
	(median 80)	(median 73)	(median 80)		(median 170)	(median 160)	(median 176)	(median190)	
Blood loss (ml)	25 ± 7	24 ± 10	25 ± 7	0.42	70 ± 61	91 ± 75	69 ± 54	36 ± 26	<0.01
Resected specimen weight (g)	55.82 ± 32.6	56.8 ± 38.3	55.7 ± 32.1	0.91	348 ± 227	401 ± 343	333 ± 160	309 ± 125	0.04
Hospital day	3.7 ± 0.8	3.7 ± 0.8	3.7 ± 0.8	0.85	5.9 ± 1.6	5.2 ± 1.7	6 ± 1.4	6.6 ± 1.6	<0.01
Margin involved				1					0.52
Yes	8 (4.1)	1 (4.8)	6 (4)		3 (1.2)	0 (0)	2(1.6)	1 (1.9)	
No	186 (95.9)	20 (95.2)	166 (96)		253 (98.8)	76 (100)	126(98.4)	51 (98.1)	
Follow-up (month)	31.1 ± 29.4	14.7 ± 26.6	36.7 ± 28.8	<0.01	52.2 ± 31.8	68.1 ± 27.3	52.1 ± 33.4	26.1 ± 14	<0.01
	median 24.8	median 7.3	median 31		median 49.9	median 81.7	median 54.1	median 29.9	
Recurrence				1					0.44
Yes	6 (3)	1 (4.2)	5 (2.8)		20 (7.4)	8 (7.1)	12(9)	2 (3.6)	
No	196 (97)	23 (95.8)	173 (97.2)		252 (92.6)	76 (92.9)	121(91)	53 (96.4)	
CD score				0.92					0.14
0	186 (92.1)	22 (91.7)	164 (92.1)		196 (72.1)	67 (79.8)	88 (66.2)	41 (74.6)	
I	15 (7.4)	2 (8.3)	13 (7.3)		61(22.4)	13 (15.5)	34 (25.6)	14 (25.5)	
II	1 (0.5)	0 (0)	1 (0.6)		10 (3.7)	2 (2.4)	8 (6)	0 (0)	
III b	0 (0)	0 (0)	0 (0)		5 (1.8)	2 (2.4)	3 (2.3)	0 (0)	
**Nipple ischemia necrosis grading**									0.22
No ischemia-Gr 0					214 (78.7)	73 (86.9)	97 (72.9)	44 (80)	
Transient ischemia, recovered without volume loss-Gr 1					23 (8.5)	4 (4.8)	13 (9.8)	6 (10.9)	
Partial necrosis of nipple with partial volume loss-Gr 2					33 (12.1)	7 (8.3)	21 (15.8)	5 (9.1)	
Total necrosis of nipple with total volume loss-Gr 3					2 (7.4)	0 (0)	2 (1.5)	0 (0)	

*Excluding partial breast reconstruction cases, BCS, breast conserving surgery; NSM, nipple sparing mastectomy; IGBR, immediate gel implant breast reconstruction; C-NSM, conventional NSM; E-NSM, endoscopic assisted NSM; R-NSM, robotic assisted NSM; CD score, Clavien–Dindo score for classification of severity of complications; Gr, grade.

### Development of Various Minimal Access Techniques of NSM

The amount and type of different NSMs performed during 2011–2020 are shown in **Figure 3B**. Initially, conventional NSM (C-NSM) was dominant, but dual-axillary-areolar-incision 2D endoscopic assisted NSM (E-NSM) became the predominant minimal access mastectomy during 2011–2014. In May 2014, we started using single-axillary-incision hybrid technique of E-NSM, and in March 2017, R-NSM operations were initiated (**Figures 2**, **3B**). Single-port three-dimensional (3D) videoscope-assisted E-NSM was initiated in August 2018 and has become the main procedure of our NSM. As shown in **Figure 3C**, minimal access NSM gradually surpassed C-NSM and became the dominant procedures in the past decade.

### Operation Time, Blood Loss, and Hospital Stay of Various NSMs

The wound length of C-NSM, E-NSM, and RNSM is 9.4 ± 4.6 (median 8) cm, 5 ± 0.8 (median 5) cm, and 4.1 ± 0.8 (median 4) cm (*p* < 0.01, **Figure 2**), respectively. The mean operation time for C-NSM and IGBR was 183 ± 101 (median 160) min, E-NSM and IGBR was 208 ± 94 (median 176) min, and R-NSM and IGBR was 191 ± 64 (median 190) min (*p* = 0.12, **Table 2**). Robotic NSM (36 ± 25 ml) and E-NSM (69 ± 54 ml) have less blood loss compared with C-NSM (91 ± 75 ml, *p* < 0.01). However, longer hospital stay was observed in the E-NSM (6 ± 1.4 days) and R-NSM (6.6 ± 1.6 days) group compared with C-NSM (5.2 ± 1.7 days, *p* < 0.01). Comparison between various groups of NSMs (**Table 2**) revealed no statistical difference in complications and nipple ischemia-related events, except that dual-areolar-axillary incision NSM had higher (22.8%) grade II+III nipple ischemia-necrosis compared with single axillary incision NSM (9.4%).

### Operation Time Change and Overall Cost of Various NSMs

**Figure 4:**
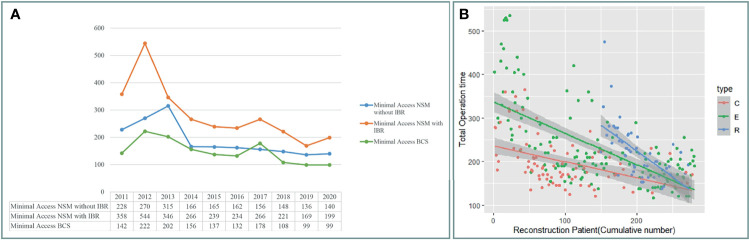
Operation time change after cases accumulation. **(A)** Following case accumulations over the past decade, various minimal access breast surgeries decreased operation time gradually. **(B)** The operation time of unilateral nipple sparing mastectomy with immediate gel implant breast reconstruction for conventional NSM (C-NSM), endoscopic assisted NSM (E-NSM), and robotic assisted NSM (R-NSM). The operation of each procedure performed was plotted in a timeline sequence, and all the operation time of three different types of NSM decreased and gradually merged together. C, C-NSM; E, E-NEM; R, R-NSM.

**Figure 5:**
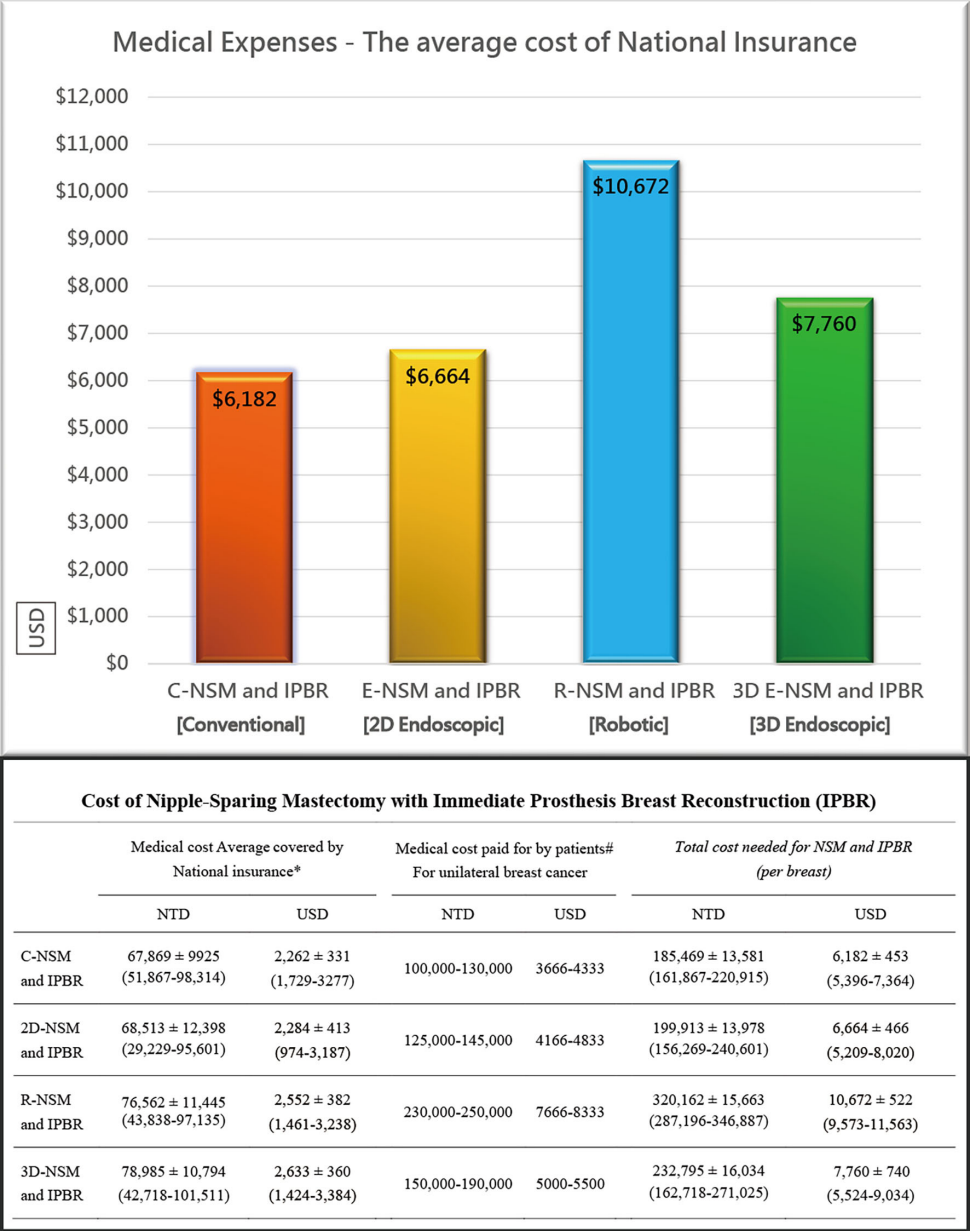
Cost analysis of various nipple sparing mastectomy (NSM) and immediate prothesis implant breast reconstruction (IPBR). With the introduction of two-dimensional endoscopic assisted NSM (2D E-NSM) retraction-type technique, the cost increased slightly from 6,182 [conventional NSM (C-NSM)] to 6,664 USD (about 500 USD more). Shift to robotic assisted NSM (R-NSM) dramatically increased medical cost to 10,672 USD (about 4,000 USD more). Inspired from R-NSM, we started the single-port 3D endoscopic assisted NSM (E-NSM) program in August 2018, and quickly this procedure became popular due to balanced clinical outcome and medical cost (7,760 USD), in which nearly 3,000 USD could be saved when compared with R-NSM. Following the advances of various minimal access breast surgeries, the overall cost also increased parallelly. R-NSM almost doubled the cost of C-NSM, and by adopting 3D E-NSM, the cost decreased near 3,000 USD without compromising the cosmetic result.

**Figure 4A** shows a decrease in operation time over the 10 years of experiences with BCS and NSM with and without breast reconstructions. The operation time of C-NSM, E-NSM, and R-NSM is plotted against cumulative patient number (**Figure 4B**), which reflects that as technique matures, the operation time for these three procedures all decreased and eventually merged without apparent difference (*p* = 0.12) The overall cost of C-NSM and IGBR (6,182 ± 453 USDs), 2D E-NSM and IGBR (6,664 ± 466 USDs), R-NSM and IGBR (10,672 ± 522 USDs), and single-port 3D E-NSM and IGBR (7,760 ± 740 USDs) is summarized and shown in **Figure 5**. R-NSM was significantly associated with higher cost than other NSMs (*p* < 0.01).

### Oncologic Outcome of Conventional Versus Endoscopic or Robotic Assisted NSM

The positive margin rate was 0% in C-NSM, 1.6% in E-NSM, and 1.9% in R-NSM (*p* = 0.52). The median follow-up duration of NSM patients was 49.9 months. The local recurrence (**Table 2**), disease-free survival, and overall survival were not statistically different between C-NSM and minimal access (endoscopic or robotic assisted) NSMs (**Supplementary Figure S2**). In multivariate analysis for disease recurrence, minimal access breast surgery was not associated with increased disease recurrence risk (**Supplementary Table S1**).

## Discussion

In the current study, we report the development and trend of MABS (EABS or RABS) for surgical treatment of breast cancer in the past 10 years from single surgeon at one institute. The proportion of breast cancer patients who received MABS (EABS or RABS) increased gradually (**Figure 3A**). The highlight of MABS is its small and inconspicuous operation wound (**Figure 2**). This value is more prominent when mastectomy is inevitable. When mastectomy is done *via* MABS, we can pursue an aesthetic scar-less mastectomy. With case accumulation, the overall operation time of various MABS decreased gradually (**Figure 4**).

BCS had become the mainstream for small early breast cancer, and our previous publication on the application of endoscopic (4, 38) or robotic (19) assisted techniques in BCS has shown promising results. Adopting E-BCS successfully decreased 23% of median wound length (**Table 2**) with incisional wound hidden in inconspicuous location (**Supplementary Figure 1**). We had applied robotic assisted technique in some breast cancer patients receiving BCS; these patients are mainly for partial breast reconstructions with either robotic assisted harvest of LDF (19) or omentum flap (23). From **Figure 1**, the major applications of robotic surgery in breast surgeries were total skin and NAC preserving mastectomy (R-NSM) (27) (**Figure 2**). The application of RABS in BCS alone, however, was not recommended due to high cost and long operation time.

NSM yields better cosmetic outcome and has proven oncologic safety; hence, it is increasingly used in breast cancer patients indicated for mastectomy (39, 40). Mastectomy usually requires larger incisional wound, by adopting E-NSM or R-NSM, about 46.8% to 57.5% of wound length was spared (**Table 2**), approaching the goal of aesthetic scar-less mastectomy (**Figure 2**) (35). Compared with about 23% reduction of BCS wound length, the value of MABS was more apparent in NSM, where scar size is nearly halved (**Figures 1**–**3**). This positive impact had been reflected in our previous patients-reported-outcome studies (9, 17): satisfaction rates of R-NSM (92% excellent, 8% good) and E-NSM (87.5% excellent, 12.5% good) were significantly higher than C-NSM (75.6% excellent, 24.4% good). Current optimal scar incision location had been shifted from inner breast to the anterior axillary line at the level of NAC. This location enables sentinel lymph node biopsy and NSM with IGBR. An asset of this location is that this scar could be well hidden along the bra-line (**Figure 2**).

Initially, the E-NSM was developed with non-gas inflation retraction type endoscope (5, 13, 41), and for vein harvest from posterior dissection (separation of pectoralis major and breast glandular tissue, **Figure 2**). Then, a semi-areolar incision was made for anterior skin flap dissection using optical port trocar with tunneling method. This dual-areolar-axillary-incision is well-adapted for small- to medium-sized breast, patients without breast reconstruction, and patients receiving tissue expander breast reconstruction. However, for direct gel implant breast reconstruction and large-sized breast patients, NAC ischemia/necrosis risk would be increased (4). The shift from initial dual-areolar-axillary-incision to single-axillary-incision in 2D E-NSM (13, 16) greatly decreased the rate of NAC ischemia/necrosis (Gr II+III) from 22.8% (1.4% total necrosis rate) to 9.4% (0.5% total necrosis rate, *p* < 0.01). However, this transition also increased operation difficulty and related operation time (**Figure 4**).

The development of R-NSM successfully solved the difficulties faced by single-axillary-incision 2D E-NSM (7, 8, 27, 34, 42) through better visual acuity with 3D videoscope and wristed articular robotic arms (**Figures 2**, **3B**). The major drawback of R-NSM is the high cost, which is an extra 4,000 USD more (**Figure 5**) compared to C-NSM (17). Inspired from R-NSM, we started performing single-port 3D endoscopic assisted NSM (E-NSM) in August 2018 (**Figure 3B**) (31). This procedure became widely accepted due to balanced clinical outcome and medical cost (7,760 USD) (15), nearly 3,000 USD could be saved when compared with R-NSM (**Figure 5**). As medical cost remained an important consideration, we believe that R-NSM can work as a bridge and help surgeons be familiar with techniques of advanced endoscopic breast surgery such as single-port 3D E-NSM (15).

Extended operation time is one criticism of MABS (5, 6, 28, 29), especially towards R-NSM (29). **Figure 4A** showed that in the past 10 years, operation time had decreased dramatically. When we compare the first vs. last 10 cases of E-NSM and R-NSM, a reduction of 51.5% and 32% operation time was observed respectively (**Figure 4B**). Surgical length of MABS has now decreased to acceptable duration after much accumulated case (33), making surgeons more experienced and able to standardize the performed procedures (27) (**Figure 4**). From our perspective, air inflation system would be the predominant system in E-NSM (15, 16, 31) and R-NSM (8, 27, 34, 42); however, retraction system might be sufficient for E-BCS (11, 30).

The oncologic safety of E-BCS versus C-BCS was confirmed in our previous study (26) by adopting propensity score matching. Oncologic safety is not shown in the current cohort as patient-selection criteria and case number of C-BCS were quite different from E-BCS (**Table 1**). The preliminary oncologic safety of minimal access (endoscopic or robotic assisted) NSM versus C-NSM is shown in **Table 2** and **Supplementary Figure 2**, which present comparable disease-free survival and overall survival for all three procedures. In multivariate analysis for disease recurrence, minimal access breast surgery was not associated with increased recurrence risk. However, difference in various groups and relative short follow-up time in R-NSM should be noted.

Our current study is limited in its retrospective nature, limited case numbers, and single-surgeon experience Nonetheless, data were collected prospectively over a 10-year-period, and the results derived from this study are solid and reliable. The observed trend of increasing MABS performed in early breast cancer and shortening of operation time in various MABS with case accumulated experience could be extrapolated to other centers in the world.

## Conclusion

The era of minimal access (endoscopic and robotic assisted) breast surgery is approaching as an increasing number of hospitals around the world adopt these minimal access breast surgical techniques in the treatment of breast cancer. The highlight of MABS lies in its small inconspicuous operation scar and high patient satisfaction. The previously criticized extensive operation time had decreased steadily due to technique refinements and the surgical team’s procedural familiarity. The cost of minimal access mastectomy increased from conventional to E-NSM and nearly doubled in R-NSM. The newly developed single-port 3D E-NSM, which worked just as well as R-NSM while greatly decreased medical cost, could be an alternative choice.

## Data Availability Statement

The data analyzed in this study are subject to the following licenses/restrictions: The dataset could be available only by asking the principal investigator, and will be released after permission. Requests to access these datasets should be directed to hwlai650420@yahoo.com.tw.

## Ethics Statement

The studies involving human participants were reviewed and approved by Institutional Review Board of the Changhua Christian Hospital. The patients/participants provided their written informed consent to participate in this study. Written informed consent was obtained from the individual(s) for the publication of any potentially identifiable images or data included in this article.

## Author Contributions

Study design: H-WL and D-RC. Data collection: S-TC and S-LL. Data analysis: Y-JL and S-JK. Manuscript: H-WL and D-RC. All authors contributed to the article and approved the submitted version.

## Funding

This study was funded by the Ministry of Science and Technology of Taiwan, and the number of this funding was MOST 110-2314-B-371-009-. This study was also sponsored by research funding provided by the Changhua Christian Hospital 109-CCH-IRP-093 and 110-CCH-IRP-042. We also received research funding from Intuitive Surgery CRG09-08232019.

## Conflict of Interest

The authors declare that the research was conducted in the absence of any commercial or financial relationships that could be construed as a potential conflict of interest.

## Publisher’s Note

All claims expressed in this article are solely those of the authors and do not necessarily represent those of their affiliated organizations, or those of the publisher, the editors and the reviewers. Any product that may be evaluated in this article, or claim that may be made by its manufacturer, is not guaranteed or endorsed by the publisher.

## Supplementary Material

Supplementary Figure 1 Various operation photos. C: conventional breast surgery, E, endoscopic assisted breast surgery; PM, partial mastectomy; TM, total mastectomy.

Supplementary Figure 2 Survival analysis of various nipple sparing mastectomy with immediate breast reconstruction.
