# Supplementary figures and images for: Minimal Access (Endoscopic and Robotic) Breast Surgery in the Surgical Treatment of Early Breast Cancer—Trend and Clinical Outcome From a Single-Surgeon Experience Over 10 Years
Source: Front Oncol. 2021 Nov 19;11:739144. doi: 10.3389/fonc.2021.739144 (PMC8640170; doi:10.3389/fonc.2021.739144)

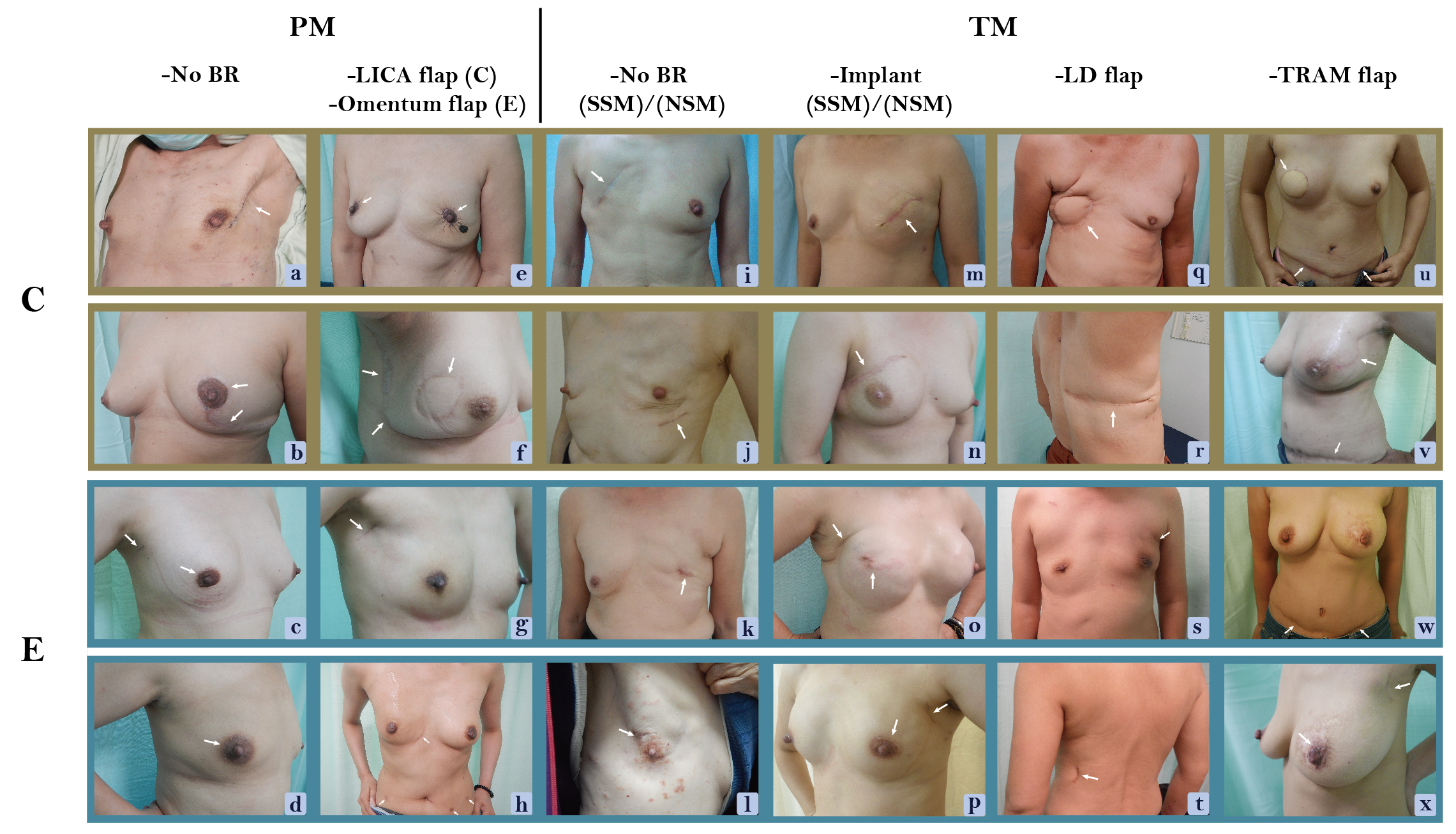

Supplement: Supplementary Figure 1 — Various operation photos. C: conventional breast surgery, E, endoscopic assisted breast surgery; PM, partial mastectomy; TM, total mastectomy. [file Image_1.jpeg]
