# Supplementary material for: Minimal Access (Endoscopic and Robotic) Breast Surgery in the Surgical Treatment of Early Breast Cancer—Trend and Clinical Outcome From a Single-Surgeon Experience Over 10 Years
Source: Front Oncol. 2021 Nov 19;11:739144. doi: 10.3389/fonc.2021.739144 (PMC8640170; doi:10.3389/fonc.2021.739144)
Supplement: Supplementary Figure 2 — Survival analysis of various nipple sparing mastectomy with immediate breast reconstruction. [file Presentation_1.pptx]

## Slide 1
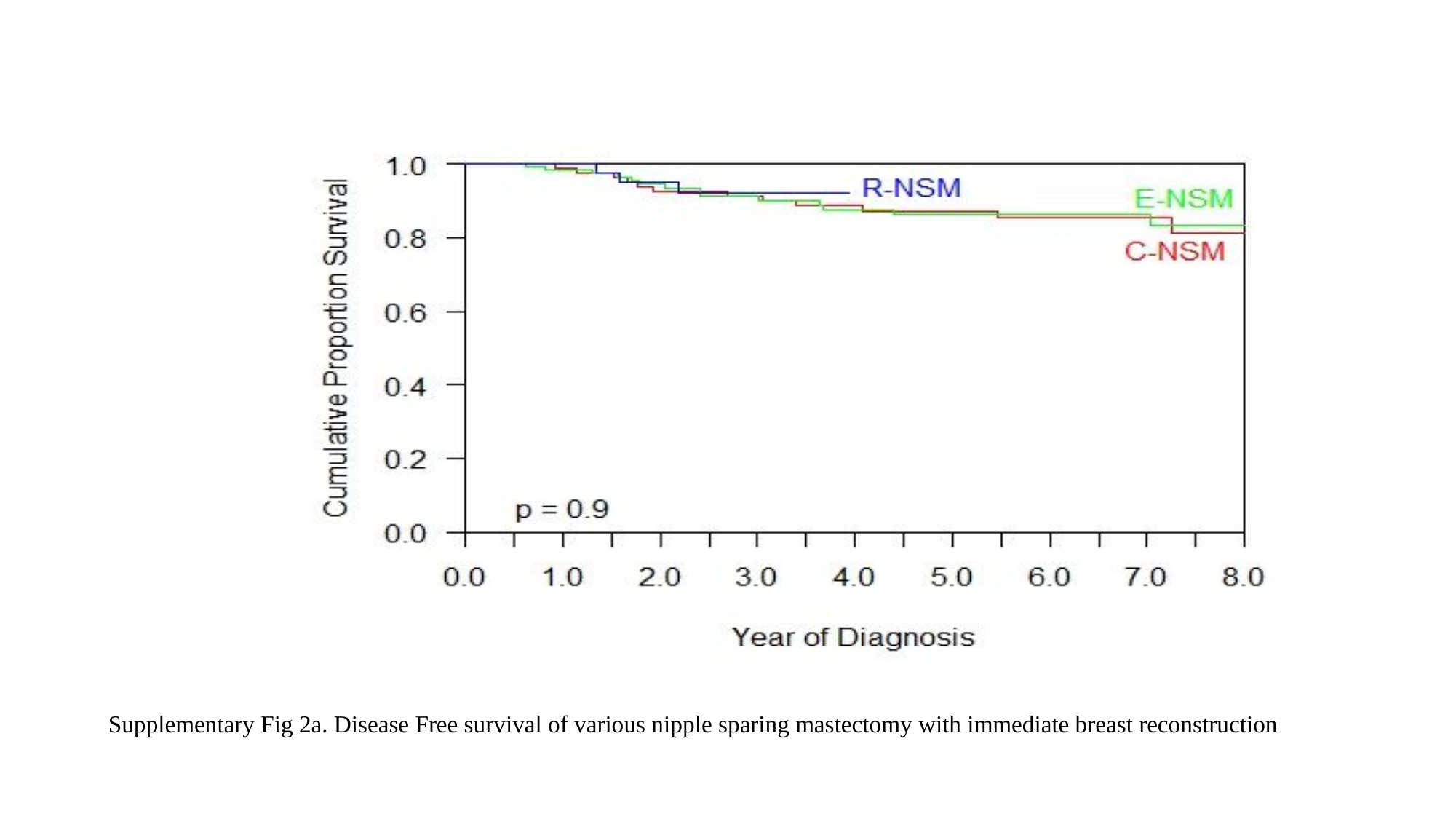

Supplementary Fig 2a. Disease Free survival of various nipple sparing mastectomy with immediate breast reconstruction

## Slide 2
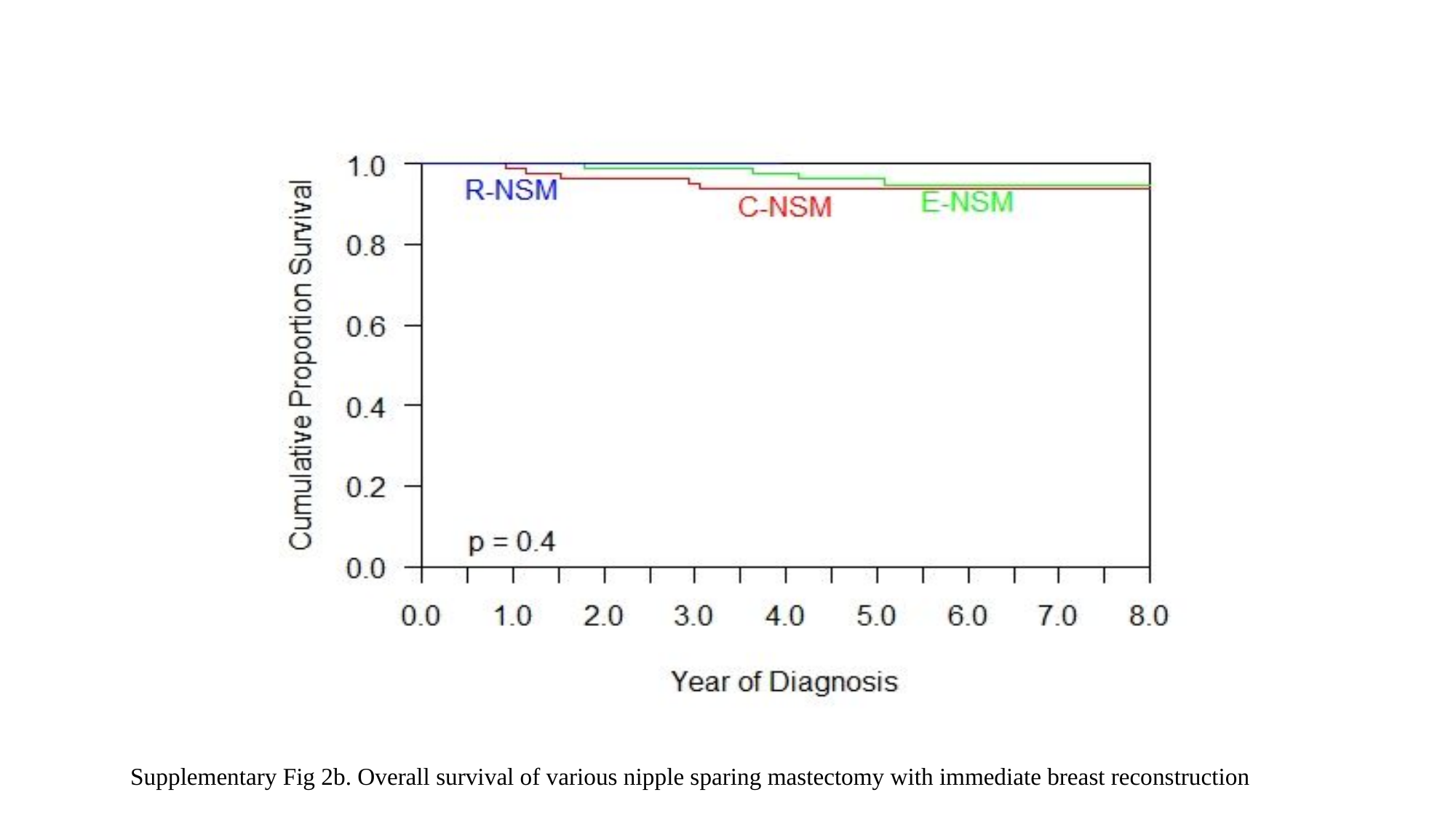

Supplementary Fig 2b. Overall survival of various nipple sparing mastectomy with immediate breast reconstruction
