# Supplementary material for: Minimal Access (Endoscopic and Robotic) Breast Surgery in the Surgical Treatment of Early Breast Cancer—Trend and Clinical Outcome From a Single-Surgeon Experience Over 10 Years
Source: Front Oncol. 2021 Nov 19;11:739144. doi: 10.3389/fonc.2021.739144 (PMC8640170; doi:10.3389/fonc.2021.739144)
Supplement: Supplementary file 3 [file Table_1.docx]

Supplementary Table 1. Cox hazard for risk factors of disease recurrence

|  | Univariate analysis | | | Multivariate analysis | | |
| --- | --- | --- | --- | --- | --- | --- |
| Parameters | Odds ratio | 95% CI | p | Odds ratio | 95% CI | p |
| Age | 1.01 | 0.99~1.03 | 0.3 |  |  |  |
| Pathology Tumor size (cm, neoadjuvant cases excluded) | 1.179 | 1.12~1.23 | 0<0.01* | 1.186 | 1.10~1.28 | 0<0.01* |
| Lymph node metastasis | 1.607 | 1.12~-1.24 | 0<0.01* | 1.138 | 0.83~1.56 | 0.42 |
| Grade | 2.187 | 1.54~3.11 | 0<0.01* | 1.403 | 0.84~2.36 | 0.20 |
| ER(positive) | 0.453 | 0.29~0.71 | 0<0.01* | 0.812 | 0.45~1.47 | 0.49 |
| PR(positive) | 0.575 | 0.37~0.88 | 0.012 |  |  |  |
| Her2(positive) | 1.789 | 1.16~2.77 | 0<0.01* | 1.466 | 0.87~5.47 | 0.15 |
| Ki-67(>20% vs <=20) | 2.392 | 1.52~3.77 | 0<0.01* | 1.186 | 0.65~2.18 | 0.58 |
| Operative method (mastectomy vs BCS) | 1.949 | 1.04~3.67 | 0.03 |  |  |  |
| Operative method (Minimal Access vs Conventional Breast Surgery) | 0.385 | 0.25~0.58 | 0<0.01* | 0.647 | 0.39~1.08 | 0.09 |
| Margin status(Yes) | 2.459 | 1.00~6.08 | 0.05 |  |  |  |
| Neoadjuvant therapy(Yes) | 1.88 | 0.91~3.9 | 0.09 |  |  |  |
